# Cannonball jellyfish digestion: an insight into the lipolytic enzymes of the digestive system

**DOI:** 10.7717/peerj.9794

**Published:** 2020-09-08

**Authors:** Raul B. Martínez-Pérez, Jorge A. Rodríguez, Luis Alonso Leyva Soto, Pablo Gortáres-Moroyoqui, Lourdes M. Diaz-Tenorio

**Affiliations:** 1 Departamento de Biotecnología y Ciencias Alimentarias, Instituto Tecnológico de Sonora, Ciudad Obregón, Sonora, Mexico; 2 Biotecnología Industrial, Centro de Investigación y Asistencia en Tecnología y Diseño del Estado de Jalisco A.C., Zapopan, Jalisco, Mexico; 3 Dirección de Cátedras, Consejo Nacional de Ciencia y Tecnología, Ciudad de México, Mexico

**Keywords:** Digestion, *Stomolophus* sp. 2, Lypolitic activity, Lipase, Phospholipase, Gastric pouch, Sonoran coast, Cannonball jellyfish

## Abstract

The digestive system and metabolism of the cannonball jellyfish *Stomolophus* sp. 2 are not well-known. The digestion study was critical to explain its ecology and bloom success. Different enzymes are involved in food digestion, which hydrolyze carbohydrates, proteins, and lipids. This study detected lipolytic activity in enzymatic extracts from gastric pouches of *Stomolophus* sp. 2 collected in the summer of 2013 at Bahía de Kino, Sonora, México (28°47′47″N 111°57′25″W). Lipase/esterase activity showed optimal pH at 11.0 and 50–60 °C with a half-life (t_1/2_) of 33 min at 55 °C, whereas halotolerance of this activity was recorded from 0-4 M NaCl. Metal ions Ca^2+^ and Mn^2+^ did not affect the activity, but Mg^2+^ decreased it 14.2% ± 3.15, while chelating agents as ethylenediaminetetraacetic acid reduced the activity 8.55% ± 2.13. Inhibition of lipase/esterase activity with tetrahydrolipstatin and paraoxon-ethyl decreased the activity 18.2% ± 2.3, and 62.80% ± 0.74, respectively, whereas phenylmethanesulfonyl fluoride (a protease inhibitor) did not affect it. The enzyme displayed a higher specificity for short-chain triglycerides, but triolein, coconut oil, olive oil, and fish oil were hydrolyzed. For the first time, phospholipase activity from the gastric pouch of *Stomolophus* sp. 2 was detected using L-α-phosphatidylethanolamine from chicken egg yolk as a substrate. These results suggest that *Stomolophus* sp. 2 hydrolyze several kinds of lipids, and lipolytic enzymes are active at alkaline pH under different saline conditions, which may be essential to digest different preys.

## Introduction

Diverse factors, such as meteorological conditions, salinity, currents, pressure, water temperature, and predation play a significant role in jellyfish population size. Several causes have been postulated to explain blooms, such as climate change, eutrophication, commercial marine species overfishing and the creation of artificial marine structures that provide the conditions for the benthic stage of its life history (Pitt & Purcell, 2007). When environmental conditions are favorable jellyfish life cycle is promoted; first, polyp strobilation is developed (Peggy Hsieh, Leong & Rudloe, 2001; Sugiura, 1965); then, the specimen is transformed to ephyra stage; finally, a jellyfish is released and grows to adult stage. Jellyfish population is characterized by significant changes in abundance. When a bloom occurs, eggs and larvae of pelagic species are preyed by jellyfish, which can directly reduce this species recruitment; this situation causes a considerable depletion in commercial fisheries and affects the economy of the countries that trade them (Pitt, Welsh & Condon, 2009; Ruzicka, Daly & Brodeur, 2016). The cannonball jellyfish *Stomolophu meleagris* belongs to cnidarians of the phylum *Coelenterata*, class *Scyphomedusae*, order *Rhizostomeae*, family *Stomolophidae*, and genus *Stomolophus* (Li et al., 2014). Its distribution is from New England, USA to Brazil in the Atlantic and southern California, USA to the Equator in the Pacific Ocean. However, recent studies in *Stomolopus* genus from the Gulf of California have classified the cannonball as *Stomolophus* sp. 2 (Getino Mamet, Gómez Daglio & García-De León, 2019; Gómez Daglio & Dawson, 2017). The cannonball jellyfish are found in waters with an average temperature of 23.1 °C (74°F) and in salinities from 17.7–36.5 parts per thousand (ppt) with an average of 33.8 ppt. Due to its abundance and recent blooms, ecological studies are needed, especially related to their role in the food web. Currently, different studies of their life cycle and ecology have been performed, mainly those related to morphology, growth and recruitment characteristics (Palomares & Pauly, 2009).

Jellyfish are considered carnivorous organisms. In scyphozoans, digestion involves an extracellular phase and subsequently an intracellular phase (Chuin, 1929; Yonge, 1931). In most species, the stomach contains gastric filaments (gastric cirri) that deal with extracellular digestion within the stomach, but some species can digest prey using oral arms. Polyps lack gastric cirri, but they can release enzymes concentrated on the longitudinal septa (Arai, 1997). Jellyfish feed on a variety of species, including ctenophores, siphonophores, chaetognaths, larvae, eggs, small fishes, and crustaceans (Álvarez Tello, López-Martínez & luch Cota, 2016), planktonic micro crustacea, pelagic polychaetes, and other small jellyfishes (Larson, 1976). Once the prey is captured by the jellyfish digitata, the prey passes into ciliated grooves and subsequently to the canal system extending to the stomach (Larson, 1991).

Previous studies have investigated *S. meleagris* prey preference and the duration of the digestion process. Only have a few reports been found about jellyfish digestive enzymes and how they work. For example, proteolytic and glycogenase activities were detected in scyphozo upside-down jellyfish (*Cassiopea frondosa*) although no amylase, lactase or cellulose were found (Smith, 1936) while enzymes, such as glycogenase and lipase, were found in golden medusa (*Mastigias papua*) (Ohtsuki, 1930). Another study identified trypsin activity in Portuguese man-of-war (*Physalia physalis*) and alkaline and acid phosphatase, chymotrypsin and lipase activity in cannonball jellyfish (*S. meleagris*) (Arai, 1997; González-Valdovinos, Ocampo & Tovar-Ramírez, 2019). Bodansky & Rose (1922) observed the presence of proteases and amylases in tissue suspensions of *S. meleagris* gastric cirri.

Due to the kind of prey that jellyfish eats, and the little knowledge about its digestion, biochemical identification of lipolytic enzymes (EC 3.1.1.X) is important to understand the nutrition and life cycle of jellyfish species. Lipases and esterases are key hydrolases in animal digestion.

Lipases display maximal activity towards water-insoluble long-chain triglycerides (TAGs) (Arpigny & Jaeger, 1999). Esterases hydrolyze water-soluble or emulsified esters with relatively short fatty acid chains (less than 10 carbons) while “true” lipases are generally more active toward emulsified, long-chain fatty acids (more than 10 carbon) (Brault et al., 2012); phospholipases A and B catalyze hydrolysis of fatty ester in the two-positions of 3-*sn*-phospholipid to release fatty acids and lysophospholipids (Mayer & Marshall, 1993).

Previous studies on cannonball jellyfish (polyp, ephyra, and medusa stages) have reported lipolytic activity with synthetic substrates (González-Valdovinos, Ocampo & Tovar-Ramírez, 2019), but no distinction has been made between digestive or intracellular lipases or lipolytic esterases. This study analysed the gastric organ (gastric pouches) from the adult stage of *Stomolophus* sp. 2, using synthetic and natural oils (*p*-nitrophenol esters, triglycerides, and oils) to know the capacity of lipolytic enzymes in the digestive system from jellyfish; subsequently, if in whole-body jellyfish polyp, ephyra and medusa stages the lipase activity was observed; then, if lipolytic activity found in gastric pouches from the adult phase of *Stomolophus* sp, 2 corresponded to digestive lipase/esterase enzymes.

Therefore, this study aimed to contribute to a better understanding of cannonball jellyfish lipid digestion, showing the characterization of lipolytic activities from the digestive system of the blue cannonball jellyfish *Stomolophus* sp. 2.

## Material and Methods

### Reagents

Substrates used: *p*-nitrophenyl acetate (C2), *p*-nitrophenyl butyrate (C4), *p*-nitrophenyl valerate (C5), *p*-nitrophenyl octanoate (C8), *p*-nitrophenyl decanoate (C10), *p*-nitrophenyl laurate (C12), *p*-nitrophenyl myristate (C14), *p*-nitrophenyl palmitate (C16), *p*-nitrophenyl stearate (C18), vinyl butyrate (V4), vinyl laurate (V12), tripropionin (TC3), tributyrin (TC4), trioctanoin (TC8), triolein (TC18), methylumbelliferyl-butyrate (MUF-B), L-α-phosphatidylcholine from egg yolk and fish oil; Inhibitors and detergent: tetrahydrolipstatin (THL), paraoxon**-**ethyl (E600), phenylmethanesulfonyl fluoride (PMSF), *N*-lauroylsarcosine sodium salt (NLS) and sodium dodecyl sulfate (SDS); buffers: 2-(*N*-morpholino)ethanesulfonic acid (MES), 3-morpholinopropane-1-sulfonic acid (MOPS), 2-cyclohexylamino)ethanesulfonic acid (CHES), 3-(cyclohexylamino)-1-propanesulfonic acid (CAPS). Substrates, inhibitors and buffers were purchased from Sigma-Aldrich-Fluka (St. Louis, MO, USA). Coconut oil and other reagents were obtained from local suppliers.

### Specimen collection

**Figure 1:**
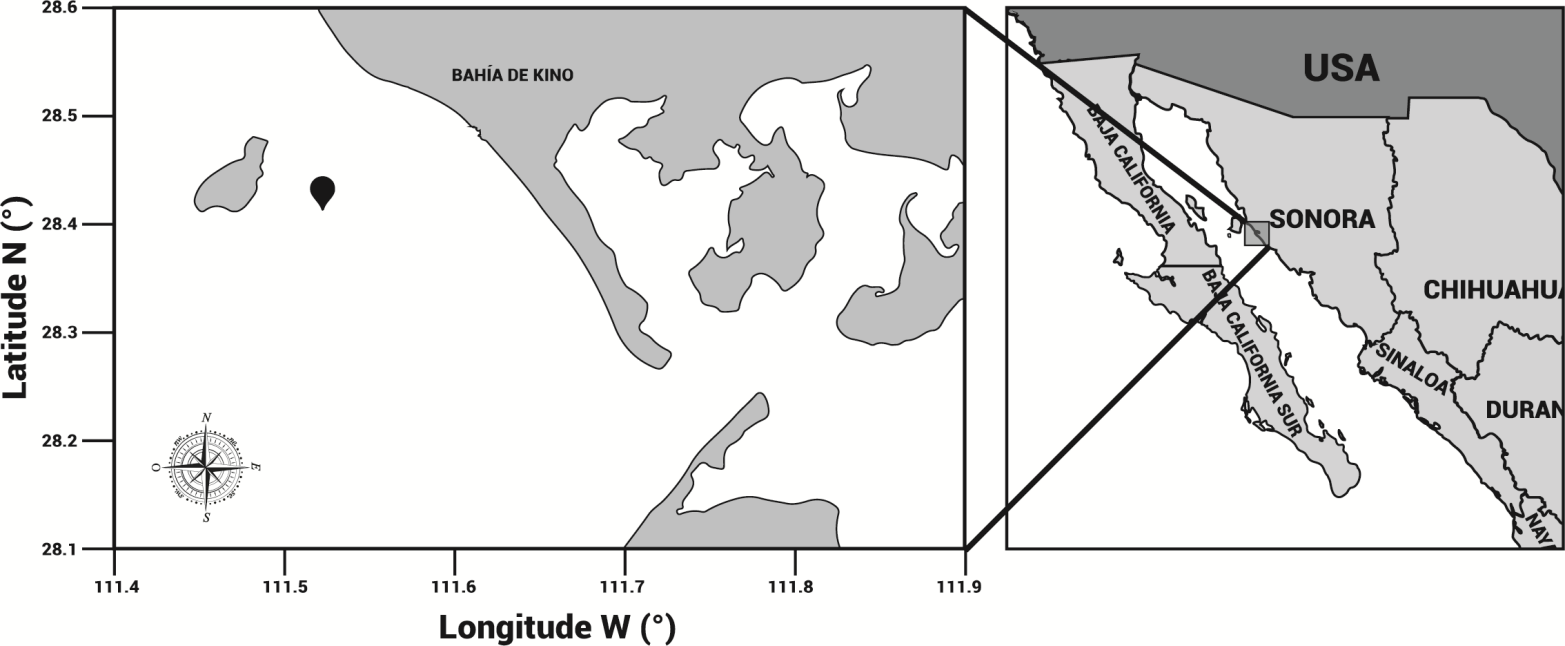
Area of study. *Stomolophus* sp. 2 sampling location in Bahía de Kino, Sonora, México in summer 2013 (28°47′47″N 111°57′25″W). Black pin locator indicates sampling site.

**Figure 2:**
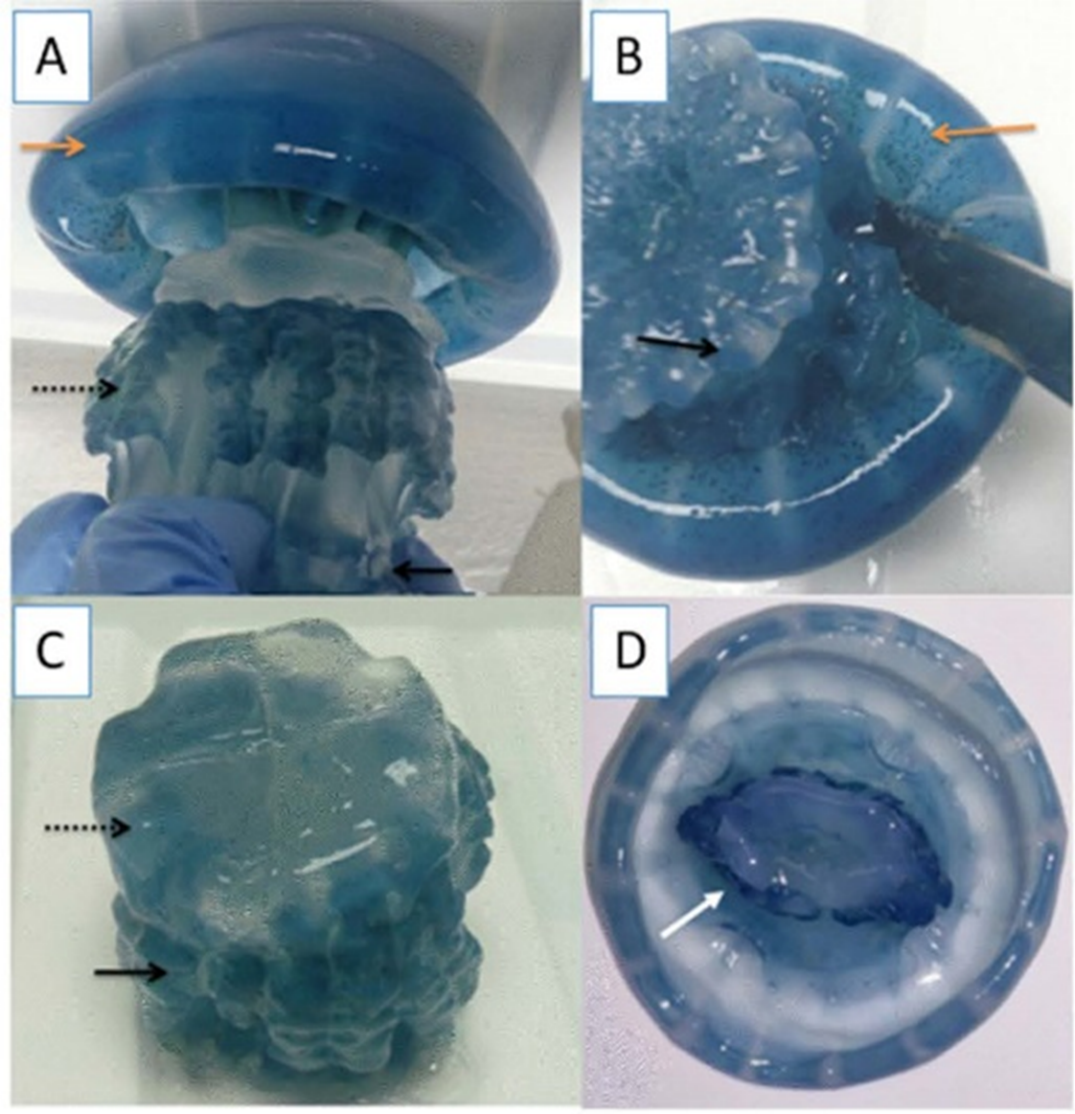
Gastric pouches obtained from *Stomolophus* sp. 2. (A) Jellyfish, black arrow indicates terminus of the oral-arms cylinder; a dotted black arrow indicates scapulet; orange arrow indicates umbrella; (B) cuts around scapulet from the umbrella; (C) separation of scapulet and oral-arms; (D) Umbrella, white arrow indicates stomach.

Specimens of cannonball jellyfish *Stomolophus* sp. 2 were collected in the spring of 2013 from surface waters at Bahía de Kino, Sonora, México (28°47′47″N 111°57′25″W) (Fig. 1), using a dip net (10-mm square mesh aperture size). Twenty adult cannonball jellyfish with an average diameter length of 87 mm and weight of 326 g were dissected to obtain digestive organs after catch. The gastric pouches were exposed by cutting around the stomach on the sub-umbrella side and removed (Fig. 2); then, the samples of each organism were maintained in 50-mL plastic tubes at 4 °C for 2 h; afterward, they were freeze-dried and stored at −20 °C for further analyses.

### Enzymatic extract of cannonball jellyfish gastric pouch

The enzymatic extract was prepared from 50 mg of freeze-dried gastric pouches from *Stomolophus* sp. 2, which were homogenized at 4 °C to low stirring speed in one mL of distilled water for 30 min; then, samples were centrifuged 10,000 *g* at 4 °C for 15 min to eliminate tissue debris. The supernatant was stored at −20 °C for further analyses.

### Spectrophotometric assay for lipase/esterase activity determination

Lipase/esterase activity was determined by a spectrophotometric assay using *p*-nitrophenyl decanoate (C10) as substrate. The release of *p*-nitrophenol was monitored at 415 nm during enzymatic hydrolysis. The reaction mixture was prepared by diluting 1:10 (v/v) a solution of 10 mM of C10 (dissolved in 2-methyl-2-butanol) with a buffer solution (50 mM Tris-HCl, pH 8.0, and 17 mM *N*-lauroylsarcosine sodium salt); 40 µL (0.88 mg/mL) of the jellyfish gastric pouch extract with 160 µL of the reaction mixture were placed in a microplate. The release of *p*-nitrophenol was monitored at 415 nm at 37 °C for 15 min and at time intervals of 30 s. The reaction rate was calculated with the absorbance curve slope versus time by using a molar extinction coefficient of 22,931 cm^−1^ M^−1^ with 17 mM *N*-lauroylsarcosine sodium salt (Mateos et al., 2007). All assays were performed in triplicate. One enzyme unit (U) was defined as the amount of enzyme releasing 1 µmol of *p*-nitrophenol per minute under the tested conditions.

### Acyl chain length specificity determination

The acyl chain length specificity towards different *p*-nitrophenyl (*p*-NP) esters was determined at 37 °C in 50 mM Tris-HCl buffer, pH 8.0. Fatty acid chain lengths from C2 to C18 were used as substrates (10 mM) dissolved in 2-methyl-2-butanol was diluted 1:10 (v/v) with buffer. When the synthetic substrate was C2, C4, and C5 the buffer solution was prepared without *N*-lauroylsarcosine sodium salt detergent—extinction molar coefficients were 22,233 cm^−1^ M^−1^ and 22,931 cm^−1^ M^−1^ for solutions with and without detergent, respectively. One unit of activity was defined as the amount of enzyme that releases 1 µmol of fatty acid per minute in the assay conditions.

### Titrimetric assay for lipase/esterase and phospholipase activity

#### Fatty acyl chain length specificity of lipase/esterase on vinyl esters

The fatty acyl chain length specificity was measured by using two different vinyl esters solutions: 73.6 mM vinyl butyrate (V4), and 35.9 mM vinyl laurate V12), both were dissolved in 2.5 mM Tris-HCl, pH 8.0 buffer. The activity was determined by titration using pH-Stat method at constant pH value (8.0) at 37 °C. The reaction was done in a glass vessel containing 82 mg of lyophilized jellyfish gastric pouch content dissolved in 10 mL of substrate. The free fatty acids released were titrated with 0.1 M NaOH (Müller-Santos et al., 2009). The activity was calculated from the curve slope and expressed as enzyme units, where one unit (U) of enzymatic activity was defined as the liberation of 1 µmol of fatty acid per minute in the assay conditions. The results were expressed as units per gram of freeze-dried jellyfish gastric pouch (U/g).

#### Lipase/esterase activity on triglycerides

Lipolytic activity was measured by titration. Free fatty acids released from TC3, TC4, TC8, TC18, olive oil, coconut oil, and fish oil emulsions were measured by adding 0.1 N NaOH on pH-Stat device at constant pH value (pH 8.0). Each assay was performed at 37 °C containing 2.5 mM Tris-HCl (pH 8.0), 150 mM NaCl, 2 mM CaCl_2_. When oils and triolein were used, the substrates were pre-emulsified with gum arabic (3% w/v) (Gargouri et al., 1984). The activity was calculated from the curve slope and expressed as enzyme units. One activity unit corresponded to 1 µmol of the fatty acids released per minute under assay conditions. The results were expressed as units per gram of freeze-dried gastric pouch (U/g).

#### Phospholipase activity and analysis of lipolysis products by thin-layer chromatography

Phospholipase activity was tested by the release of fatty acids and measured by pH-Stat (Metrohom, GPT Titrino) titration method under mechanical stirrer in a 10 mL reaction vessel at 37 °C, adding 0.1 M NaOH, using L-α-phosphatidylcholine from chicken egg yolk as substrate (Abousalham & Verger, 2000). Hydrolysis products of L-α-phosphatidylcholine from chicken egg yolk at different incubation times (8, 12, and 24 h) at room temperature were analyzed directly by performing one-dimensional thin-layer chromatography (TLC). A double elution by two successive developments was performed: (1) chloroform-methanol-acetic acid-0.9% NaCl (50:25:8:4, v/v/v/v) and dried in air for 30 min; and (2) petroleum ether-ethyl ether-acetic acid (85:15:2, v/v/v) and dried in air for 30 min. Then, the plates were sprayed with 3% cupric acetate (w/v), 8% phosphoric acid (v/v) solution and heated at 160 °C for 20 min (Bjerve, Daae & Bremer, 1974).

#### Protein determination and sodium dodecyl sulfate polyacrylamide gel electrophoresis

Protein concentration was measured using bovine serum albumin as the standard (Bradford, 1976). Protein profiles of jellyfish gastric pouch extract from each organism (*n* = 20) and a mixture of them were resolved by sodium dodecyl sulfate polyacrylamide gel electrophoresis (SDS-PAGE), containing 12% acrylamide under non-reducing conditions (Laemmli, 1970). The samples were diluted with a sample buffer (0.125 M Tris-HCl, 4% SDS, 20% v/v glycerol, 0.02% bromophenol blue at pH 6.8). Electrophoretic separation was performed at 4 °C and 7 mA constant current. Then, the SDS-PAGE was stained with silver nitrate (Merrill & Washart, 1998).

### Zymographic analysis

Zymograms were performed using the fluorogenic substrate methylumbelliferyl-butyrate (MUF-B) (Prim et al., 2003). After protein separation by SDS-PAGE, the gel was incubated with a buffer solution containing 50 mM Tris-HCl at pH 8.0, 2.5% Triton X-100 (w/v) at room temperature for 30 min. Then, the gel was rinsed with deionized water and incubated in 50 mL of substrate solution (100 µM MUF-B in 50 mM Tris-HCl at pH 8.0); after incubation time (10 min), fluorescence was detected using an ultraviolet (UV) transilluminator (Bio-Rad, Gel Doc XR) (Rivera-Pérez, Del Toro & García-Carreño, 2011a).

### Effect of pH, temperature, and NaCl on lipase/esterase activity and stability

Operational variables from the cannonball jellyfish lipases were measured using *p*-nitrophenyl decanoate. To evaluate the ionic strength effect on lipase activity, NaCl concentrations were adjusted (0–4 M) in the buffer solution (50 mM Tris–HCl pH 8.0) at 37 °C. To identify optimum pH, different buffers were used: 50 mM MES, pH 6.0; 50 mM MOPS, pH 7.0; 50 mM Tris-HCl, pH 8.0; 50 mM CHES, pH 9.0; 50 mM sodium carbonate-sodium bicarbonate, pH 10; 50 mM CAPS, pH 11.0 and 50 mM KCl-NaOH pH 12.0 at 37 °C. Different extinction molar coefficients were used to calculate the lipase/esterase activity, ε (773, 5,769, 22,931, 27,362, 27,355, 28,167, 26,513 cm^−1^ M^−1^, for pH 6.0, 7.0, 8.0, 9.0, 10.0, 11.0, 12.0, respectively). For optimum temperature, lipase activity was evaluated in the range from 25–80 °C for 15 min reaction at pH 8.0. To measure lipolytic enzyme thermostability, jellyfish gastric pouch extract was incubated at 25–60 °C for 60 min. The residual activity was recorded by the spectrophotometric assay as previously described.

### Effect of metal ions and inhibitors on lipase/esterase activity

The effect of various metal ions: Ca^2+^, Mn^2+^, Mg^2+^ andthe metal-chelating agent ethylenediaminetetraacetic acid (EDTA) was evaluated on lipase/esterase activity in 50 mM Tris-HCl buffer (pH 8.0) using *p*-nitrophenyl decanoate (C10) as substrate and 40 µl (0.88 mg/mL) of jellyfish gastric pouch extract. The inhibitory effect was tested with tetrahydrolipstatin (THL), paraoxon-ethyl (E600), phenylmethanesulfonyl fluoride (PMSF) on dimethyl sulfoxide. The jellyfish gastric pouch extract was incubated at 37 °C for 60 min with 1 mM of each metal ions and inhibitor solutions; after that, the lipase/esterase activity was measured.

### Statistical analysis

The results are represented as mean of three biological samples. One-way non-parametric ANOVA was used to evaluate lipase/esterase and phospholipase activity in all assays under different reaction conditions. The difference of means between pairs was resolved using confidence intervals of Fisher’s least significant difference (LSD) test with the statistical software program (SigmaPlot v. 10.0). Differences were reported as statistically significant when *p* < 0.05.

## Results

**Figure 3:**
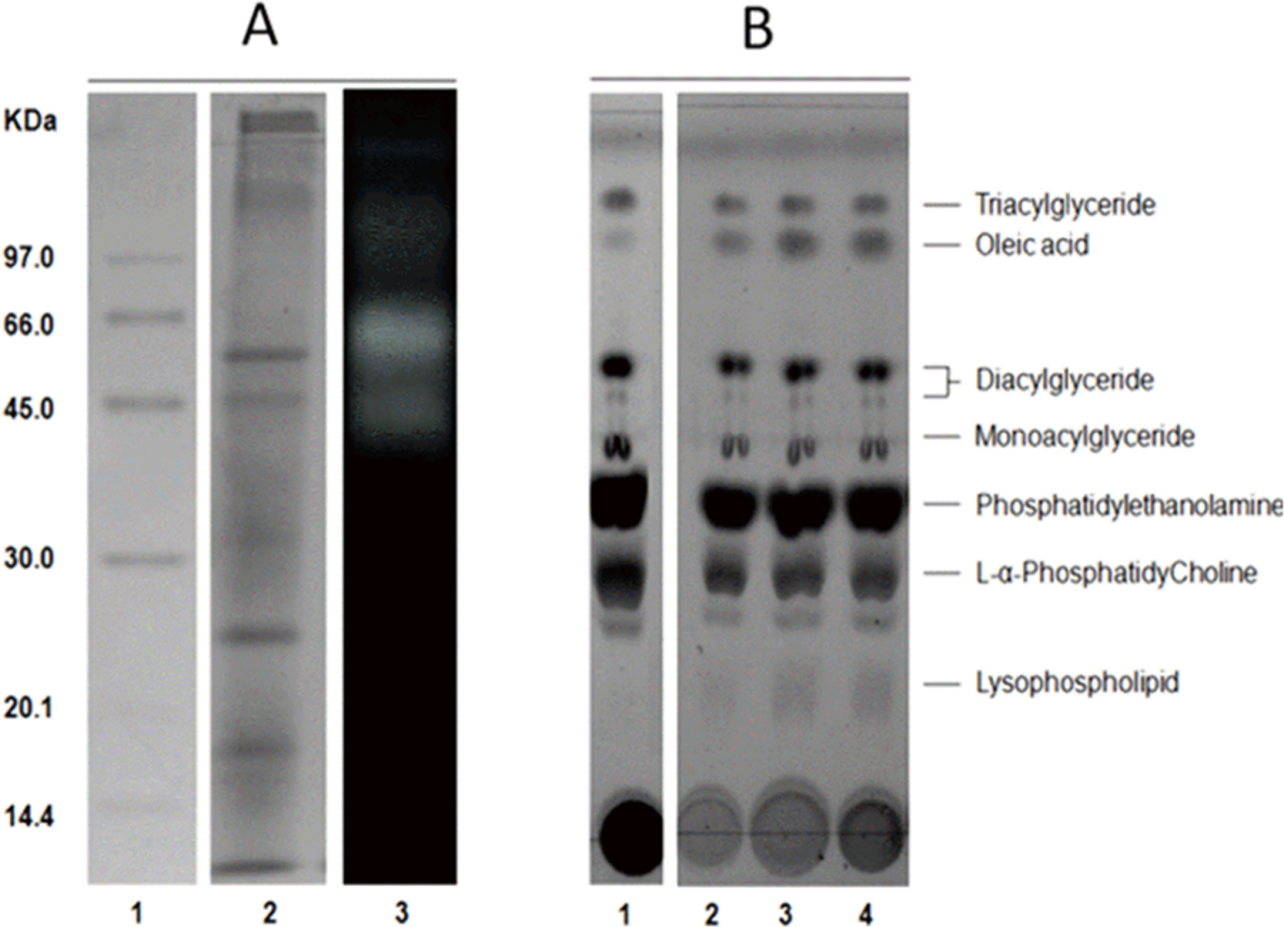
Protein profile electrophoretogram and zymogram from *Stomolophus* sp. 2 jellyfish gastric pouch extract (A). Thin-layer chromatogram of phospholipid hydrolysis products of digestive lipases from jellyfish (B). In A, molecular weight marker (1); protein profile (2); zymogram using methylumbelliferyl-butyrate (MUF-B) as substrate (3). Hydrolysis products of chicken egg yolk using the enzymatic extract from gastric pouch of *Stomolophus* sp.2 (B); blank without enzymatic extract (1); incubation with jellyfish gastric pouch extract for 8 h (2); incubation with jellyfish gastric pouch extract for 12 h (3); and incubation with jellyfish gastric pouch extract for 24 h (4).

The average protein concentration of jellyfish gastric pouch extract was 0.26 ± 0.10 of protein/mg freeze-dried gastric pouch (*n* = 20); an average of extract lipase activity from the same organisms was 0.80 ± 0.30 U/mg freeze-dried gastric pouch for *p*-nitrophenyl butyrate; and 0.23 ± 0.10 U/mg freeze-dried gastric pouch for *p*-nitrophenyl decanoate. Proteins and profiles of lipases/esterases were similar among individuals; both profiles are shown in Fig. 3A. At least three proteins hydrolzed methylumbelliferyl-butyrate (MUF-B), showing lipase/esterase activity. Figure 3B shows the separation of phospholipid hydrolysis products by thin-layer chromatography. Lanes 2–4 hydrolysis of jellyfish gastric pouch lipases are shown at different incubation times. Compared with control, lane 1 (lipid composition from chicken egg yolk) revealed the presence of lysophospholipid and free fatty acids released from L-α-phosphatidylcholine. Over time, an increase in lysophospholipid concentration was produced, which was the evidence of phospholipase activity in gastric pouch extract. To our knowledge, this is the first report of phospholipase activity in gastric pouches of the class Scyphozoa.

**Figure 4:**
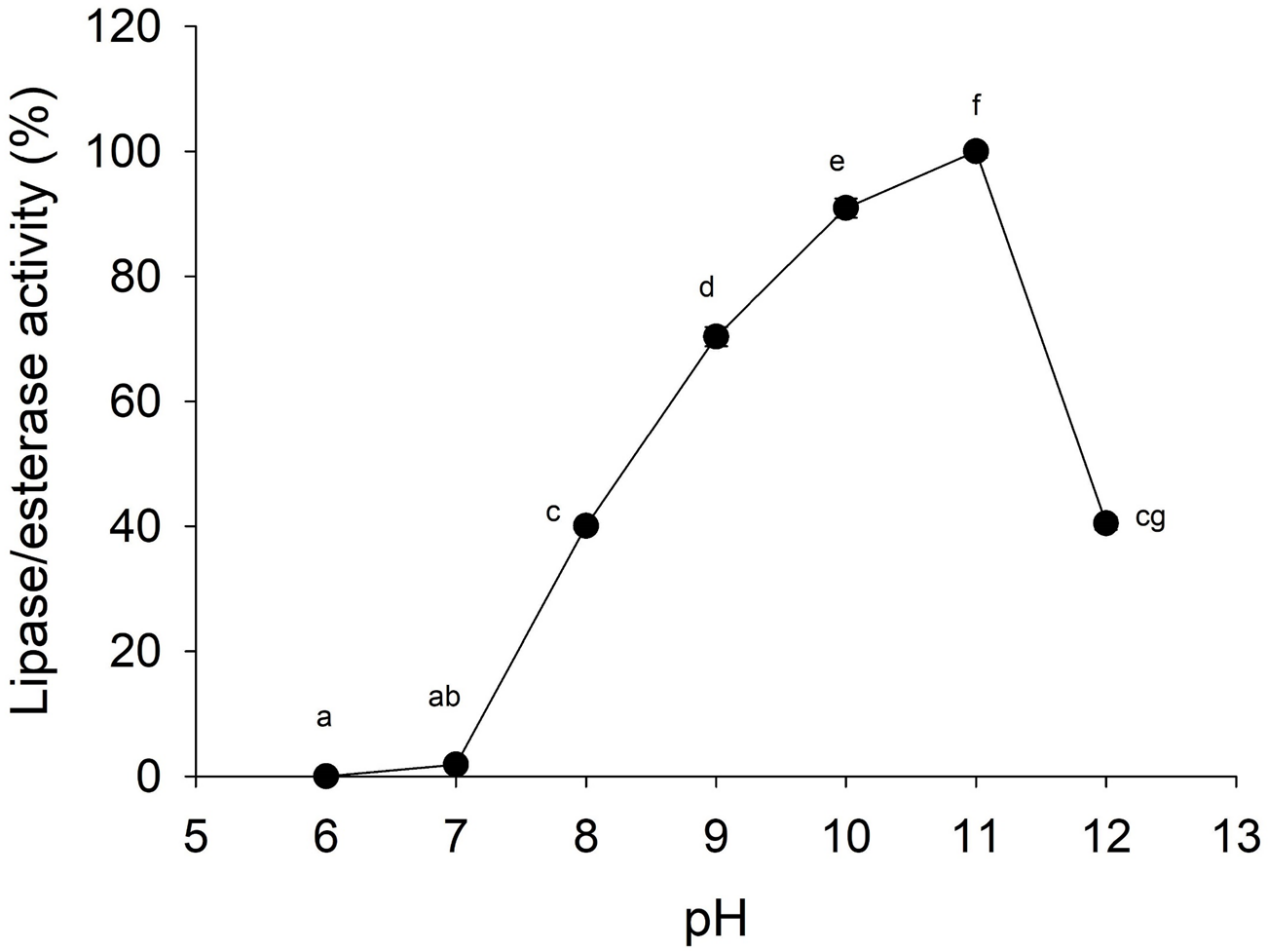
Effect of pH on lipase/esterase activity of *Stomolophus* sp. 2 jellyfish gastric pouch extract. Activity was measured with 10 mM of *p*-nitrophenyl decanoate at 37 °C. Each value represents the mean ± standard deviation (SD) of three replicate assays. Activity profiles were expressed in relation to the maximum value (=100%). Different letters indicate significant differences.

Lipolytic activity was tested in a wide range of pH (6.0–12.0), since pH 7.0 lipolytic activity had risen until it reached optimum activity at pH 11.0, and then the activity decreased (Fig. 4). At pH 6 and 7 cannonball jellyfish lipases were not able to hydrolize *p*-nitrophenyl decanoate.

**Figure 5:**
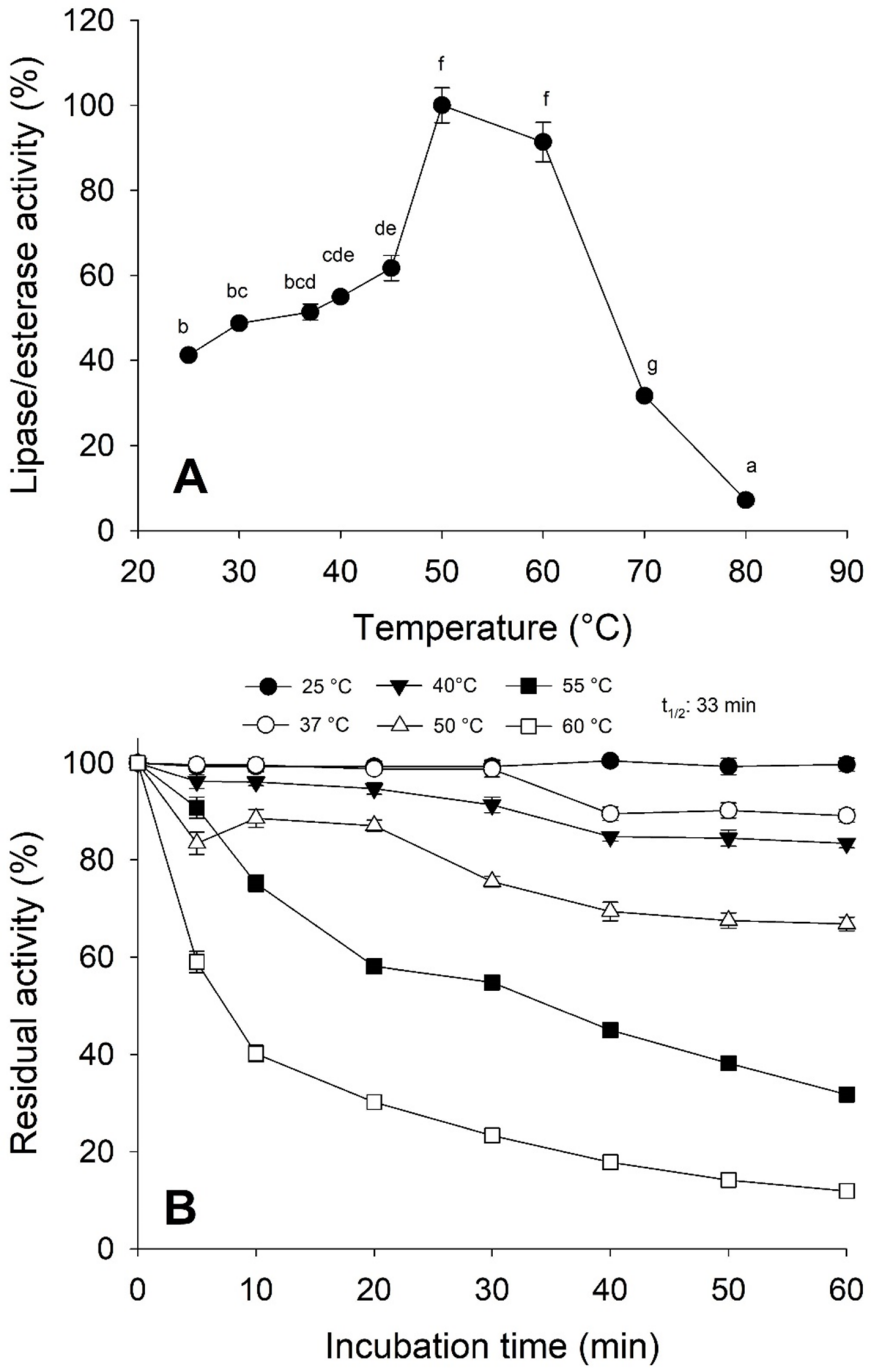
Thermal effect on lipolytic activity from *Stomolophus* sp. 2 jellyfish gastric pouch extract. (A) Effect of temperature on lipase/esterase activity of jellyfish gastric pouch extract. Each value represents the mean ± standard deviation (SD) of three replicate assays. Activity profiles were expressed in relation to the maximum value (=100%). (B) **** Thermostability of lipase/esterase activity of jellyfish gastric pouch extract. Each value represents the mean ± SD of three replicate assays. Activity profiles were expressed in relation to the maximum value (=100%). Half-life was obtained at 55 °C. In all cases, the activity was measured with 10 mM of *p*-nitrophenyl decanoate at 37 °C and pH 8.0.

Moreover, the jellyfish lipase activity displayed the optimum temperature at 50−60 °C and decreased above 60 °C (Fig. 5A). Nonetheless, optimum temperature and thermostability decreased as the temperature increased half-life (t_1∕2_) at 55 °C at 33 min (Fig. 5B).

**Figure 6:**
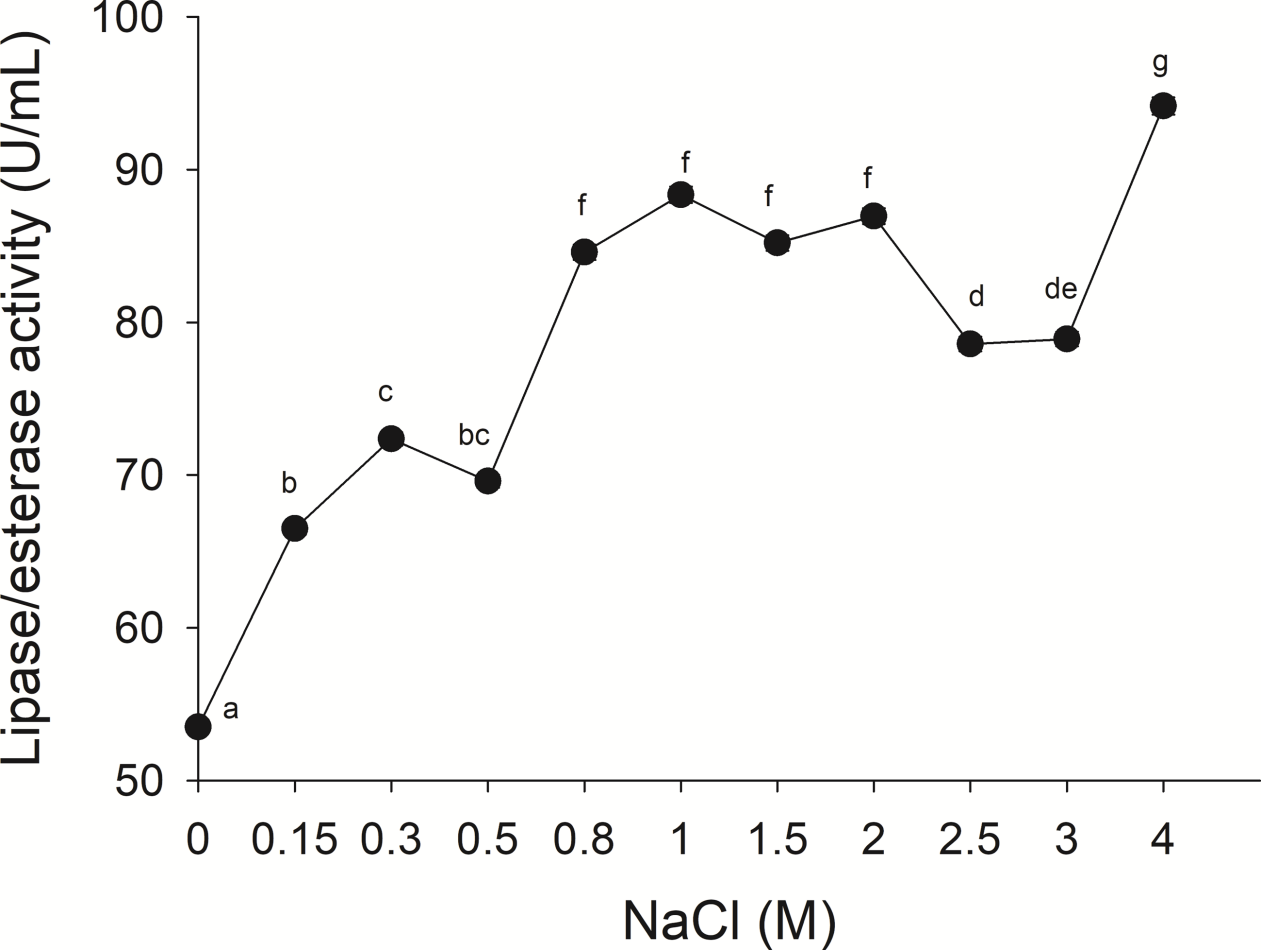
Effect of NaCl on lipase/esterase activity of *Stomolophus* sp. 2 jellyfish gastric pouch extract. Activity was measured with 10 mM of *p*-nitrophenyl decanoate at 37 °C and pH 8.0. Each value represents the mean ± standard deviation (SD) of three replicate assays. Different letters indicate significant differences.

The lipolytic activity from the gastric pouch was evaluated at different NaCl concentrations (0–4 M); the activity was influenced by ionic strength (*i*), as salt concentration increased, lipase activity also increased; a maximum value (94.16 ± 0.56 mU/mL) was observed at 4 M NaCl (*i* = 4) with respect to 53.5 ± 0.31 mU/mg at *i* = 0 (NaCl absence) (Fig. 6).

Lipolytic activity was not significantly affected by ions except for magnesium ions that reduced lipolytic activity, 15.00 ± 3.15% (Table 1). However, the paraoxon-ethyl (E600) inhibitor decreased the activity in 62.80 ± 0.74% while tetrahydrolipstatin (THL) reduced activity in 18.50 ± 2.35%. The chelating agent EDTA and lipase/esterase inhibitor phenylmethanesulfonyl fluoride (PMSF) did not show an effect in this study (Table 1).

**Figure 7:**
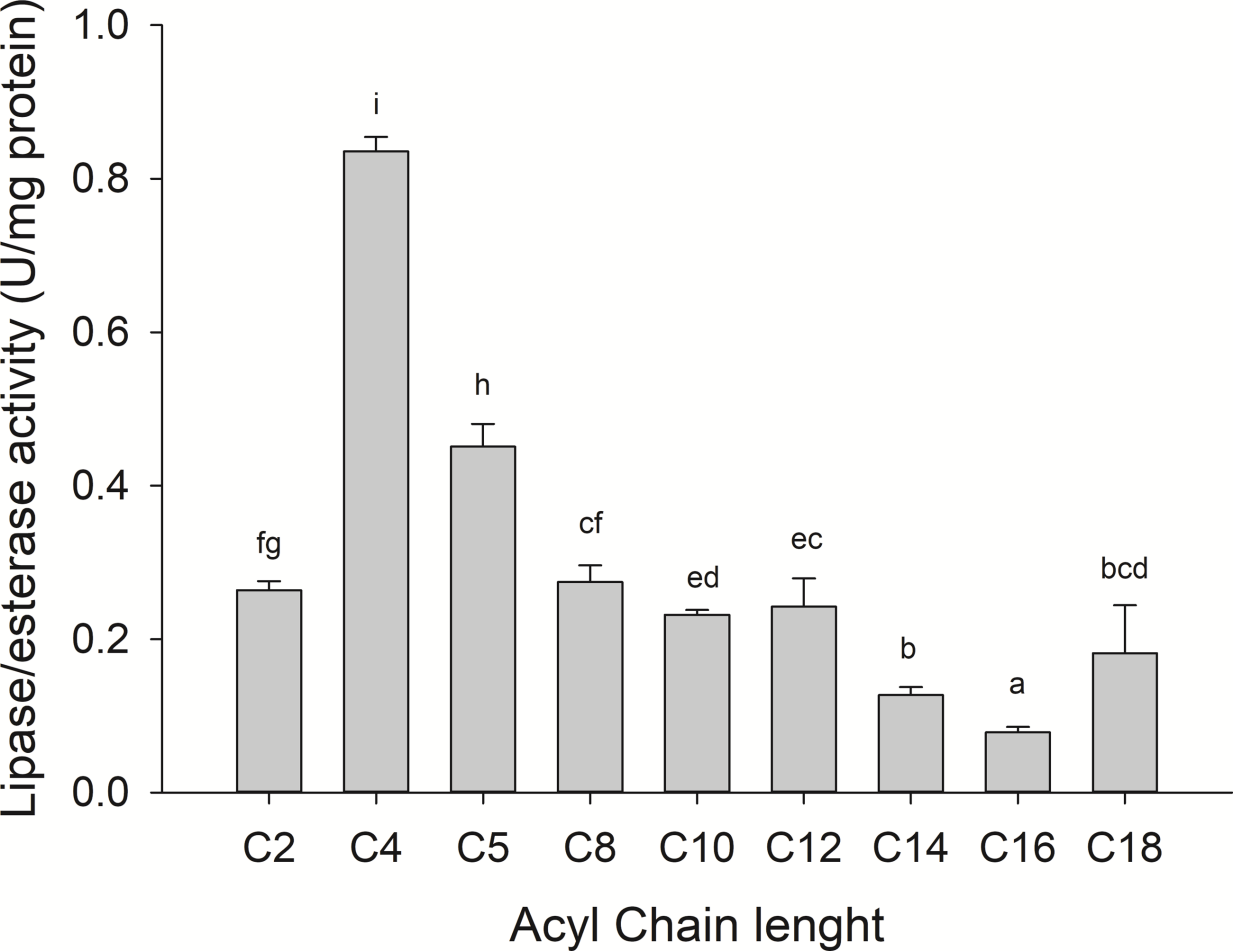
Fatty acid chain length specificity of jellyfish gastric pouch extract using *p*-nitrophenyl esters. Activity was measured at 37 °C and pH 8.0. Each value represents the mean ± standard deviation (SD) of three replicate assays. Different letters indicate significant differences.

**Figure 8:**
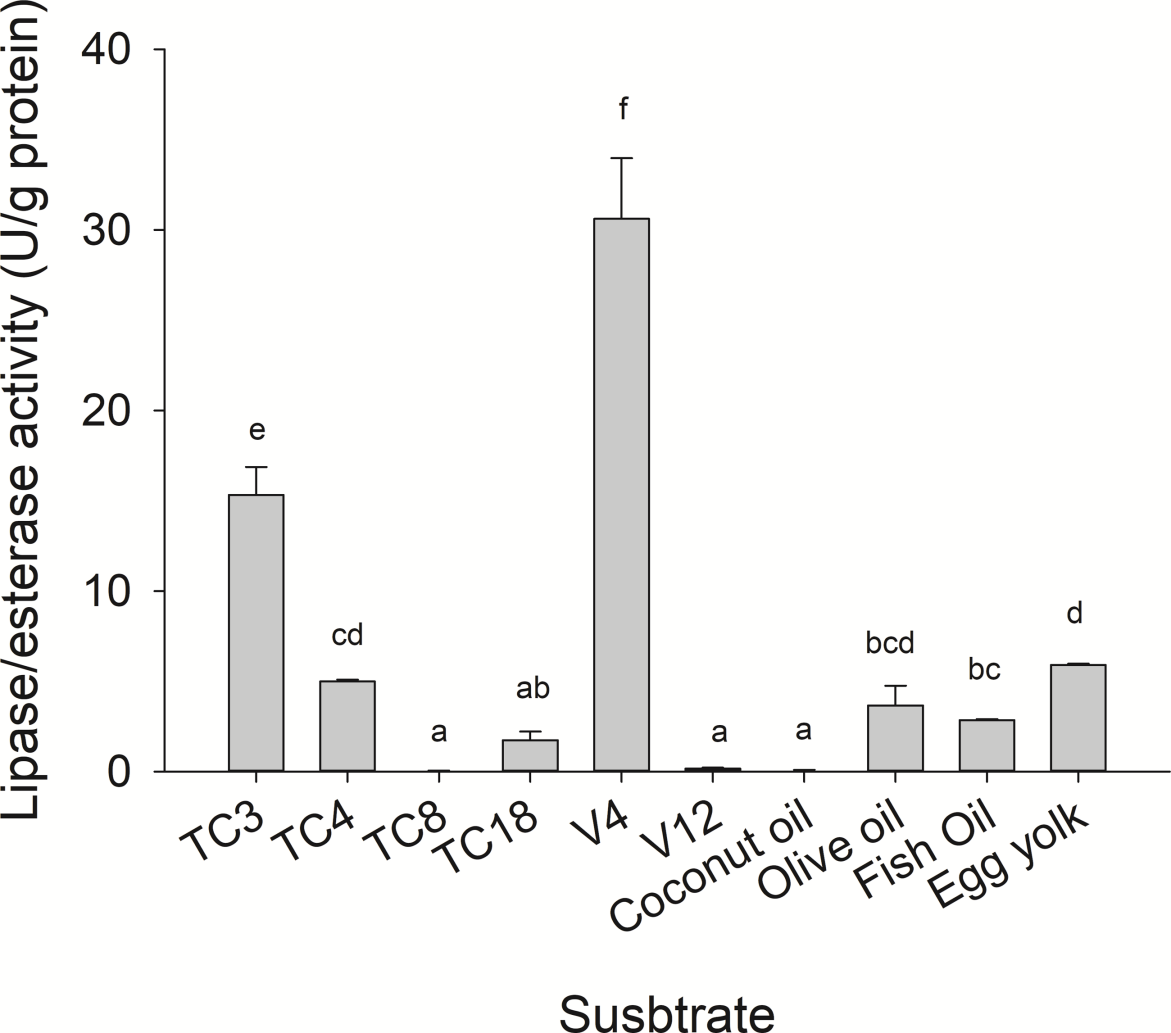
Lipase/esterase activity of *Stomolophus* sp. 2 jellyfish gastric pouch extract on triacylglycerols, phospholipids and, vinyl esters. Triacylglycerols (TC3, tripropionin; TC4, tributyrin; TC8, trioctanoin; TC18, triolein), vinyl esters (V4, vinyl butyrate; V12, vinyl laurate) and oils (olive, coconut, and fish). Phospholipase activity was measured using chicken egg yolk as substrate. For TC18 and oils, Arabic gum (3%) and for chicken egg yolk, 20 mM sodium taurodeoxycholate were used as emulsifiers. Activity was measured using freeze-dried powder. Each value represents the mean ± standard deviation (SD) of three replicate assays. Different letters indicate significant differences.

Substrate specificity was evaluated using *p*-nitrophenyl (*p*-NP) esters as substrates with different acyl chain lengths. Lipase activity for short-chain acyl substrates as *p*-NP acetate (C2), *p*-NP butyrate (C4), and *p*-NP valerate, (C5) 0.26 ± 0.01., 0.84 ± 0.02, and 0.45 ± 0.03 U/mg of protein were shown, respectively. No statistically significant results were found among medium acyl chain substrates: *p*-nitrophenyl octanoate (0.27 ± 0.02), and *p*-nitrophenyl decanoate (0.23 ± 0.01). Meanwhile, lipolytic activity on large chain acyl substrates, as *p*-NP laurate (C12), *p*-NP myristate (C14), *p*-NP palmitate (C16), and *p*-nitrophenyl stearate (C18) were 0.24 ± 0.04, 0.13 ± 0.01, 0.08 ± 0.01 and 0.18 ± 0.06 U/mg of protein, respectively (Fig. 7) when triglyceride oils and vinyl esters were used to evaluate lipolytic activity. High specificity towards tripropionin (TC3), and vinyl butyrate (V4) (15.32 ± 1.50), and 30.60 ± 3.30 U/g protein, respectively, were observed in jellyfish gastric pouch extract. No activity was detected when coconut oil and trioctanoin (TC8) were used. However, with olive oil and fish oil, lipolytic activity reached values of 11.95 3.65 ± 1.00, and 2.84 ± 0.01 U/g protein, respectively; this result was similar compared with the synthetic substrate. Higher lipolytic activity was observed when a short acyl chain was used as a substrate instead of the long one. The same happened with the coconut oil as a substrate, which has a higher content of short and medium triglycerides compared to olive oil and fish oil (Fig. 8). Phospholipase activity was measured using L-α-phosphatidylcholine from chicken egg yolk as substrate; the activity recorded was 5.91 ± 0.01 U/g protein (Fig. 8).

## Discussion

Medusae are a diverse group of planktonic predators occurring in all marine habitats. Their success is somewhat based on their ability to grow rapidly; thus, they were tightly coupled to ephemeral secondary production (Larson, 1991). Their digestive enzymes secreted in gastric pouches play an essential role in prey digestion. This research studied wild individuals; thus, differences in protein concentration and lipase/esterase activity among individuals could have been the result of many factors, such as size, reproductive stage, and feeding (Carvalho-Saucedo et al., 2009; Lucas, 1994). Plankton (microalgae, fish eggs, and larvae) is crucial for cnidarian feeding (Orejas et al., 2002; Purcell & Arai, 2001); its biochemical composition varies, but lipids are always present (Ríos et al., 1998).

Lipases from the studied jellyfish showed activity in the range from pH 8–12 with an optimum pH at 11 (Fig. 4). Similar pH optima have not been reported for any other jellyfish; the only similar pH profile has been reported for the extracellular lipase from *Pseudomonas aeruginosa* (Karadzic et al., 2006) and recombinant lipase from *Bacillus licheniformis* (Nthangeni et al., 2001). Lipolytic enzymes in the digestive system of *S. meleagris* were observed using ethyl butyrate (a non-specific substrate for true lipases) as substrate and reported as lipase (Bodansky & Rose, 1922). In marine invertebrates, as *Carcinus maenas* and *Litopenaeus vannamei*, the lipase activity described, so far, has functioned at pH 8.0 (Rivera-Perez, 2015). However, it does not happen with lipase/esterase from cannonball jellyfish *Stomolophus* sp. 2, and it is an unusual characteristic of marine lipases.

**Table 1:** Effect of different ions, metal-chelating agent, and inhibitors on lipase/esterase activity of *Stomolophus* sp. 2. jellyfish gastric pouch extract. Activity was measured with *p*-nitrophenyl decanoate at 37 C and pH 8.0 for 60 min incubation at room temperature. Each value represents the mean and standard deviation (%), of three replicate assays. Activity profiles were expressed in relation to the maximum value (=100%). Metal ions, inhibitors and chelating agent were used at 1 mM concentration; inhibitors were dissolved in dimethyl sulfoxide (DMSO) and distilled water. Distilled water (control); ethylenediaminetetraacetic acid (EDTA) and inhibitors: phenylmethanesulfonyl fluoride (PMSF), paraoxon-ethyl (E600), and tetrahydrolipstatin (THL). Different letters in the same column indicate significant differences.

Reagent (1 mM)	Activity (%, ±SD)	
Control	100.0 ± 2.2^a^	
CaCl_2_	98.6 ± 2.5^ac^	
MnCl_2_	94.5 ± 2.4^ac^	
MgCl_2_	85.8 ± 3.1^b^	
EDTA (Metal-chelating agent)	91.4 ± 2.1^bc^	
PMSF	101.2 ± 1.8^af^	
E600	37.1 ± 0.7^d^	
THL	81.4 ± 2.3^e^	

Temperature is an important factor in enzyme catalysis because protein structure can be affected and influence the thermodynamics reaction. As shown in Fig. 5A, the optimal temperature for lipase/esterase activity was from 50−60 °C, beyond this temperature, the activity was lost. In other marine invertebrates, as crab *Carcinus mediterraneus,* lipolitic activity was similar (50 °C) (Cherif et al., 2007) but not in Japanese flying squid (*Todarodes pacificus*)*,* flying squid (*Ommastrephes bartramii*) and marine sponge (*Halicona simulans*) which showed an optimal activity from 35–40, 25 and 40 °C, respectively. The thermostability results indicated that lipolytic activity reduced rapidly at 60 °C although at 25 °C it was maintained at 100% for one hour whereas at 50 °C, 33.8% of the activity was lost (Fig. 5B). This data shows that enzymes are thermostable at natural conditions where the jellyfish feeds.

No information was available about the effect of ionic strength on Scyphomedusae digestive lipolytic activity. In this study, lipase activity increased as ionic strength increased, which suggests that lipolytic enzymes are halotolerant because they show activity under high salt concentration (1–4 M) and in absence of salt, halotolerant enzymes remain active over a broad range of NaCl concentration without any specific salt dependence (Graziano & Merlino, 2014; Mevarech, Frolow & Gloss, 2000). Negatively charged amino acid residues in protein might be found in the solvent-exposed surface of the protein, as in other halophilic proteins (Fukuchi et al., 2003; Graziano & Merlino, 2014) and *Staphylococcus* sp. lipase (Daoud et al., 2013). The coastal lagoon “Las Guasimas” (Sonora, México) where the studied jellyfish species inhabits, salinity varies from 35 to 41 ppt (Padilla-Serrato et al., 2013). Jellyfish are osmoconformers; thus, if salinity is modified while swimming, then internal ionic strength changes. This halophilic characteristic of digestive lipase/esterases is critical because this specie is capable of digesting fat and oils from prey under different salt concentrations.

Lipolytic enzymes related to metal ions in this study were not dependent on Ca^+2^ because 100% of the activity was maintained without Ca^+2^, and the activity for 1 mM CaCl_2_ was 98.5% ± 2.5. In vertebrates, cationic cofactors are required for lipase activation; however, it was not observed in most invertebrates, including the white leg shrimp (*Litopenaeus vannamei*) (Rivera-Pérez, García-Carreño & Saborowski, 2011b) and Pacific flying squid (*Todarodes pacificus*) (Park, Soon-Yeong & Suk-Jung, 2008). Nonetheless, a decrease of the activity in *Haliclona simulans* (49.9% ± 0.1) was reported with Mg^2+^ (Selvin et al., 2012). In this study, a significant decrease in activity was shown only with Mg^2+^ (Table 1).

Irreversible inhibitors E600 and PMSF, and THL (reversible inhibitor of pancreatic lipases and reversible in microbial lipases) have been recognized as serine lipase inhibitors since they react with the catalytic serine residue; E600 inhibited 62.80 ± 0.74% and THL only 18.57 ± 2.35% (Table 1). PMSF did not inhibit lipolytic activity under the same conditions, probably because the active site of lipases was accessible only when the enzyme was bound to interfaces (Moulin et al., 1989); similar results were obtained with lipase from *Rhizomucor miehei* (Fig. S1). In *Bacillus subtilis* 168, lipolytic activity was strongly inhibited with 0.1 mM PMSF where a possible explanation was the absence of the hydrophobic lid; when no lipid substrate is found, the lid covers the catalytic site of many lipases (Lesuisse, Schanck & Colson, 1993). Lipase activity was not affected by the metal-chelating agent EDTA.

Lipolytic digestive enzymes from jellyfish (*Stomolophus* sp. 2) gastric pouches were able to hydrolyze short-chain triacylglycerides better than long ones, in contrast to other marine invertebrates as the whiteleg shrimp (*Litopenaeus vannamei*) digestive lipase (PVL) with 475.8 ± 4.9 U/mg protein for tributyrin and 1,787 ± 7.9 U/mg protein for triolein (Rivera-Pérez, García-Carreño & Saborowski, 2011b). In the crab digestive lipase (*Carcinus mediterraneus*), the activity was four times higher on TC4 compared to that of olive oil (Cherif et al., 2007). The tendency of digestive lipase/esterase from jellyfish to hydrolize short-chain triacylglycerides was similar to digestive lipase from marine snail (*Hexaplex trunculus*) with 400 U/mg protein for tributyrin and 100 U/mg protein for olive oil (Zarai et al., 2012). In addition, lipase/esterase activity from *Stomolophus* sp. 2 gastric pouch was similar to lipase activity from the hepatopancreas of cuttlefish (*Sepia officinalis*) (30.0 ± 0.3 U/g fresh tissue) and octopus (*Octopus marginatus*) (25.0 ± 1.25 U/g fresh tissue) (Smichi et al., 2012).

Phospholipase activity has been reported in different tissues of Scyphozoa (umbrella and tentacles). In cnidarian specimens, class Anthozoa, Hydrozoa, Scyphozoa, and Cubozoa, different levels of phospholipase activity have been found in tentacles and acontia, which are known by the high amount of nematocysts and whole-body extract organisms (Nevalainen et al., 2004), as well as in other invertebrates, such as honeybee (*Aphis melifera*) (Ownby et al., 1997), scorpions (*Mesobuthus tamulus* and *Pandinus imperator*) (Conde et al., 1999; Hariprasad et al., 2011), and cnidarian anemones (Nevalainen, 2008; Razpotnik et al., 2010).

However, until now, digestive phospholipase activity in gastric pouches has not been reported in jellyfish. In this study the phospholipase activity was observed in jellyfish gastric pouch extract; Fig. 3B shows the products of hydrolysis of L-α-phosphatidylcholine from chicken egg yolk. Lysophospholipid was produced after hydrolysis of L-α-phosphatidylethanolamine by the enzymatic extract from gastric pouches, which indicated the presence of phospholipase activity in *Stomolopohus* sp. 2. Chicken egg yolk is a rich source of phospholipids with an average of neutral lipids (65%), phospholipids (31%), and cholesterol (4%) (Aro et al., 2009). Padilla-Serrato et al. (2013) mentioned that fish eggs and fish larvae are an important component of the jellyfish diet, and both are an important source of phospholipids (Sargent et al., 1999). Thus, it was likely that phospholipase activity was higher in chicken egg yolk as a substrate than in olive oil and fish oil (Fig. 8) and prey preference may have been related to a number of the prey phospholipids. Nevertheless, further studies are needed.

Fish eggs and larvae and cypris larvae of barnacle are considered as jellyfish prey; their total lipid composition are neutral lipids and phospholipids, which represent 8.1–10.4% and 4.4–5.3% of total dry weight from the organisms mentioned (Holland & Walker, 1975). Likewise, some fish oils are mainly composed of polyunsaturated fatty acid (long-chain lipids, 18:1n9, and 18:2n6), and saturated lipids (16:0) (Xu et al., 1994). Therefore, jellyfish lipases could hydrolyze those lipids, in such a way that they assimilate hydrolysis products and use them accordingly to their needs.

## Conclusion

Our results revealed that the cannonball jellyfish digestive system contained at least three lipolytic enzymes that displayed activity against several short and long fatty acids, of which the short ones are their preferential substrate. Moreover, phospholipase activity was observed in the gastric pouch, which had not been reported before, as well as halotolerant characteristics of lipolytic enzymes (lipases and esterases). This study is a pioneer to understand the digestive capacity of jellyfish species to digest several lipid substrates.

##  Supplemental Information

10.7717/peerj.9794/supp-1 Supplemental Information 1 Raw data plotted to graph results from lipase analysis (1) Effect of pH on lipase/esterase activity, (2) Effect of temperature on lipase/esterase activity of jellyfish gastric pouch extract, (3) Thermostability of lipase/esterase activity of jellyfish gastric pouch extract, (4) Effect of NaCl on lipase/esterase activity of jellyfish gastric pouch extract, (5) Fatty acid chain length specificity of jellyfish gastric pouch extract using *p*-nitrophenol esters, (6) Lipase/esterase activity of jellyfish gastric pouch extract on triacylglycerols, phospholipids and vinyl esters, (7) Effect of different ions and inhibitors on lipase/esterase activity of jellyfish gastric pouch extract.

10.7717/peerj.9794/supp-2 Supplemental Information 2 Inhibition of lipolytic activity of *Stomolophus* sp. 2 jellyfish gastric pouch extract Zymograms were developed using methylumbelliferyl-butyrate (MUF-B) as substrate. LE: lipolytic enzymes from Jellyfish *Stomolophus* sp 2 gastric pouch extract; RL: *Rhizomucor miehei* lipase; T: inhibition with 1 mM tetrahydrolipstatin, E: inhibition with 1 mM paraoxon-ethyl (E600) and P: inhibition with 1 mM phenylmethanesulfonyl fluoride (PMSF). Enzymes were incubated at 37 °C for 1 h; then 12% sodium dodecyl sulfate-polyacrylamide gel electrophoresis (SDS-PAGE) was performed at 7.5 mA.

10.7717/peerj.9794/supp-3 Supplemental Information 3 Silver stain electrophoretogram of protein from individual content of jellyfish gastric pouch extract MM, Molecular Mass. 1–6= organism ID.

10.7717/peerj.9794/supp-4 Supplemental Information 4 Silver stain electrophoretogram of protein from individual content of jellyfish gastric pouch extract MM, Molecular Mass. 7–13, organism ID. 14, jellyfish gastric pouch extract pooled from 20 organisms.

10.7717/peerj.9794/supp-5 Supplemental Information 5 Coomasie blue stain electrophoretogram of lipase activity from content of jellyfish gastric pouch extract 7–13= organism ID. 14, jellyfish gastric pouch extract pooled from 20 organisms.

10.7717/peerj.9794/supp-6 Supplemental Information 6 Thin layer cromatogram of lipolysis products Imagen shows reagents, and pH/time conditions used during lipolysis.

## Additional Information and Declarations

### Data Availability

The following information was supplied regarding data availability: The raw data are available in the Supplementary Files.
