# Supplementary material for: Cannonball jellyfish digestion: an insight into the lipolytic enzymes of the digestive system
Source: PeerJ. 2020 Sep 8;8:e9794. doi: 10.7717/peerj.9794 (PMC7485504; doi:10.7717/peerj.9794)
Supplement: Supplemental Information 1 — (1) Effect of pH on lipase/esterase activity, (2) Effect of temperature on lipase/esterase activity of jellyfish gastric pouch extract, (3) Thermostability of lipase/esterase activity of jellyfish gastric pouch extract, (4) Effect of NaCl on lipase/esterase activity of jellyfish gastric pouch extract, (5) Fatty acid chain length specificity of jellyfish gastric pouch extract using p-nitrophenol esters, (6) Lipase/esterase activity of jellyfish gastric pouch extract on triacylglycerols, phospholipids and vinyl esters, (7) Effect of different ions and inhibitors on lipase/esterase activity of jellyfish gastric pouch extract. [file peerj-08-9794-s001.docx]

**Raw Data of “Cannonball jellyfish digestion: An insight into the lipolytic enzymes of the digestive system”, from Martínez-Pérez, et. al.**

**Effect of pH on lipase/esterase activity of GPS.**

| pH | Activity (%)* | DesvEstP |
| --- | --- | --- |
| 6 | 0.0000 | 0.0000 |
| 7 | 1.8458 | 0.2362 |
| 8 | 40.0124 | 0.3228 |
| 9 | 70.3671 | 1.5218 |
| 10 | 90.9269 | 1.5406 |
| 11 | 100 | 0.9949 |
| 12 | 40.4348 | 1.028 |
|  |  |  |
| *Relative lipolytc activity | |  |

**Effect of temperature on lipase/esterase activity of GPS.**

| Temp (° C) | Activity (%)* | DesvEstP |
| --- | --- | --- |
| 25 | 41.1899 | 0.8330 |
| 30 | 48.6899 | 0.9113 |
| 37 | 51.3442 | 1.8933 |
| 40 | 54.9406 | 1.0355 |
| 45 | 61.7396 | 2.9577 |
| 50 | 100 | 4.1096 |
| 60 | 91.3669 | 4.6428 |
| 70 | 31.6547 | 1.0258 |
| 80 | 7.1942 | 1.0973 |
|  |  |  |
| *Relative lipolytic activity | | |

**Thermostability of lipase/esterase activity of GPS.**

**Effect of NaCl on lipase/esterase activity of GPS.**

| NaCl | %Actividad* | DesvEstP |
| --- | --- | --- |
| 0 | 100 | 0.5973 |
| 0.15 | 124.2925 | 0.1888 |
| 0.3 | 135.256 | 0.3613 |
| 0.5 | 130.0816 | 0.3098 |
| 0.8 | 158.1137 | 1.9166 |
| 1 | 165.146 | 1.2929 |
| 1.5 | 159.2536 | 1.3132 |
| 2 | 162.5401 | 1.161 |
| 2.5 | 146.8976 | 1.161 |
| 3 | 147.5037 | 1.99 |
| 4 | 176.0173 | 2.7875 |
|  |  |  |
| *Realative lipolytic activity | | |

**Fatty acid chain length specificity of GPS using *p*-nitrophenol esters.**

| Substrate | %Act Relativa | DesvEstP |
| --- | --- | --- |
| C2 | 31.5544 | 1.424 |
| C4 | 100 | 2.2376 |
| C5 | 53.9548 | 3.5419 |
| C8 | 32.8347 | 2.6086 |
| C10 | 27.7253 | 0.7744 |
| C12 | 28.9937 | 4.4155 |
| C14 | 15.1849 | 1.2325 |
| C16 | 9.3574 | 0.8719 |
| C18 | 21.7423 | 7.4788 |
|  |  |  |
| *Relative lipolytic activity | | |

**Lipase/esterase activity of GPS on triacylglycerols, phospholipids and vinyl esters.** Triacylglycerols (TC3, tripropionin; TC4, tributyrin; TC8, trioctanoin; TC18, triolein), vinyl esters (V4, vinyl butyrate; V12, Vinyl laurate) and oils.

| Substrate | %Act Relativa | %Desv est |
| --- | --- | --- |
| TC3 | 50.0663 | 5.0428 |
| TC4 | 16.3346 | 0.3254 |
| TC8 | 0.0000 | 0.0000 |
| TC18 | 5.7106 | 1.5374 |
| V4 | 100.0000 | 10.9880 |
| V12 | 0.5312 | 0.1878 |
| Coconut oil | 0.0000 | 0.0000 |
| Olive oil | 11.9521 | 3.5783 |
| Fish Oil | 9.2960 | 0.1878 |
| Egg yolk | 19.3075 | 0.9850 |

**Effect of different ions and inhibitors on lipase/esterase activity of GPS.**

| Metal ion ( 1 mM) | Relative activity (%)* | DesvEstP |
| --- | --- | --- |
| Control | 100 | 2.2704 |
| CaCl2 | 98.5583 | 2.5805 |
| MnCl2 | 94.5232 | 2.4756 |
| MgCl2 | 85.7721 | 3.1524 |

| Inhibitor | Activity (%)* | DesvEstP |
| --- | --- | --- |
| Control | 100 | 2.2704 |
| EDTA | 91.4444 | 2.1348 |
| PMSF | 101.2707 | 1.8996 |
| E600 | 37.1972 | 0.7418 |
| THL | 81.4271 | 2.3599 |

*Relative lipolytic activity
