# Supplementary figures and images for: Cannonball jellyfish digestion: an insight into the lipolytic enzymes of the digestive system
Source: PeerJ. 2020 Sep 8;8:e9794. doi: 10.7717/peerj.9794 (PMC7485504; doi:10.7717/peerj.9794)

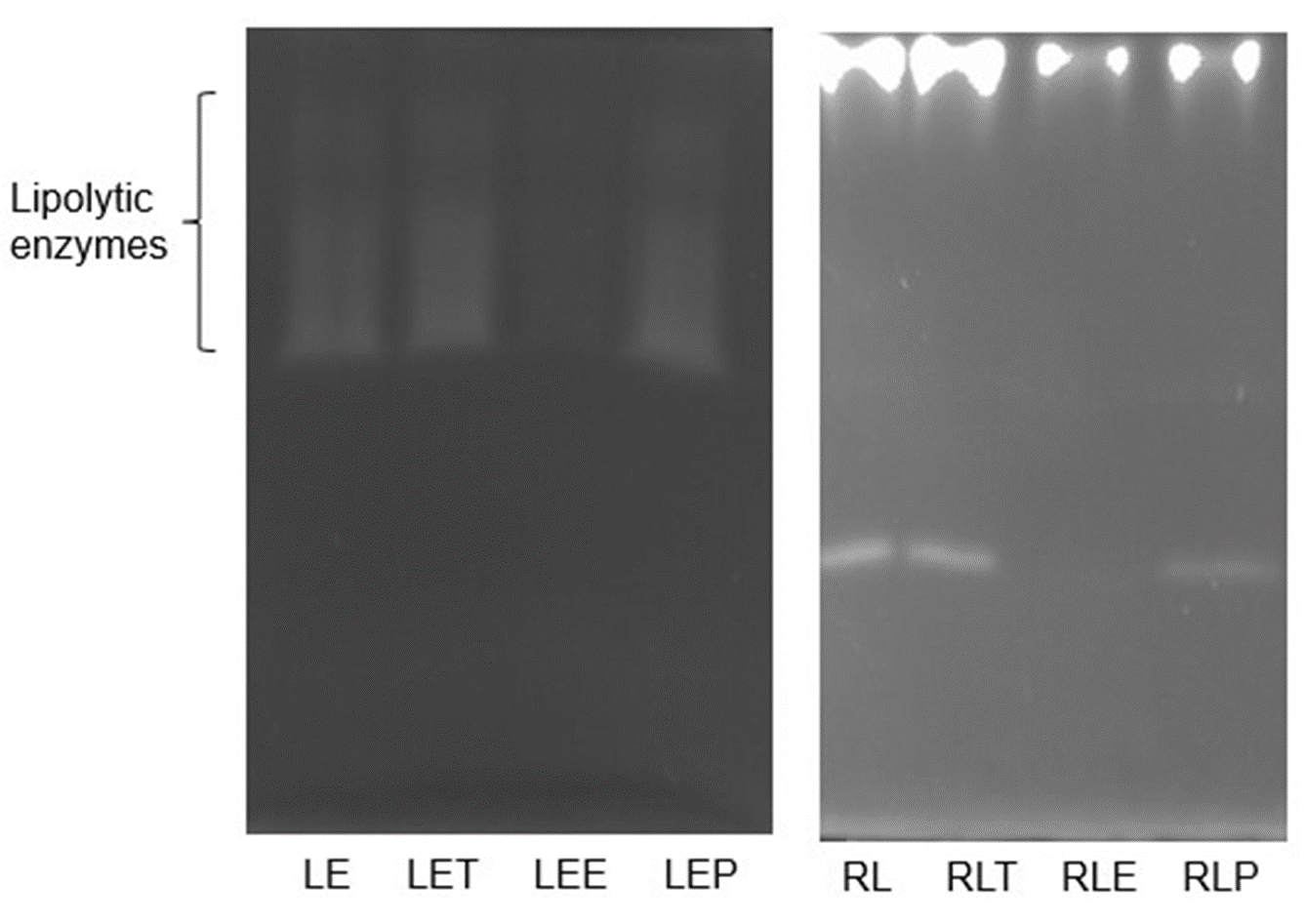

Supplement: Supplemental Information 2 — Zymograms were developed using methylumbelliferyl-butyrate (MUF-B) as substrate. LE: lipolytic enzymes from Jellyfish Stomolophus sp 2 gastric pouch extract; RL: Rhizomucor miehei lipase; T: inhibition with 1 mM tetrahydrolipstatin, E: inhibition with 1 mM paraoxon-ethyl (E600) and P: inhibition with 1 mM phenylmethanesulfonyl fluoride (PMSF). Enzymes were incubated at 37 °C for 1 h; then 12% sodium dodecyl sulfate-polyacrylamide gel electrophoresis (SDS-PAGE) was performed at 7.5 mA. [file peerj-08-9794-s002.png]

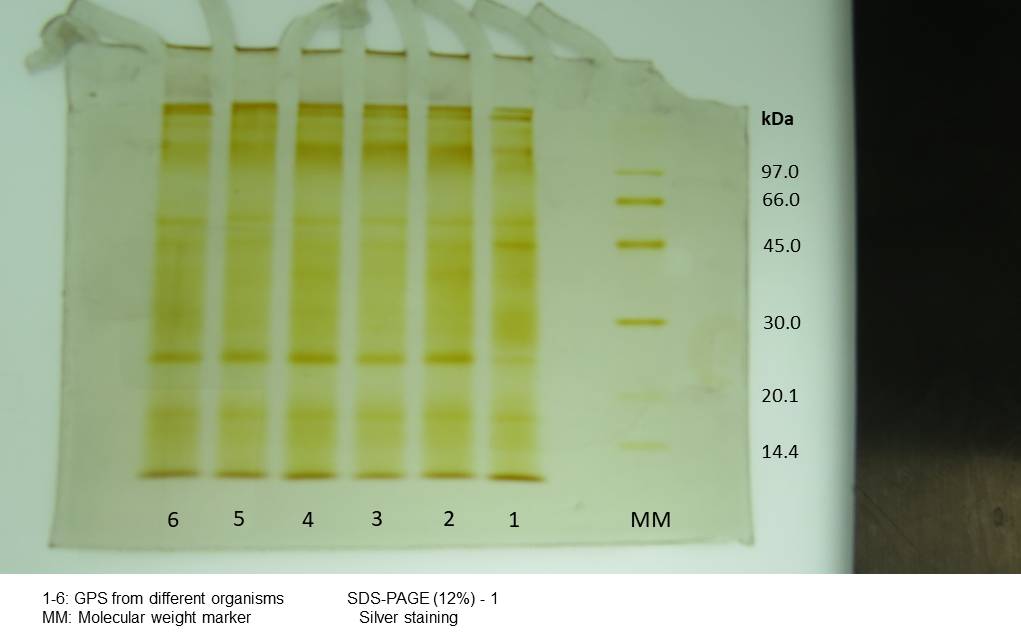

Supplement: Supplemental Information 3 — MM, Molecular Mass. 1–6= organism ID. [file peerj-08-9794-s003.png]

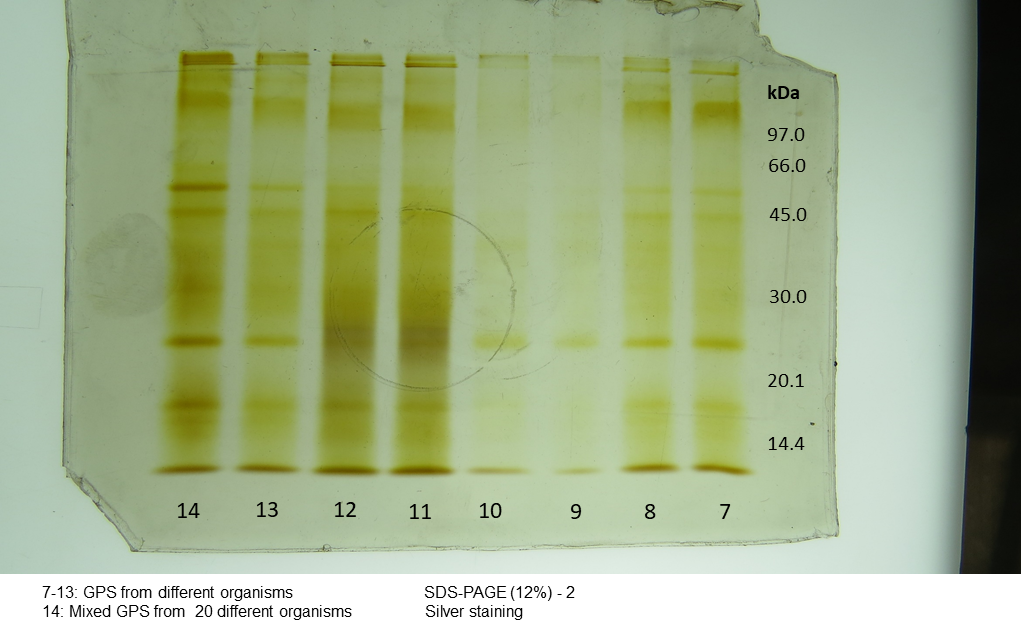

Supplement: Supplemental Information 4 — MM, Molecular Mass. 7–13, organism ID. 14, jellyfish gastric pouch extract pooled from 20 organisms. [file peerj-08-9794-s004.png]

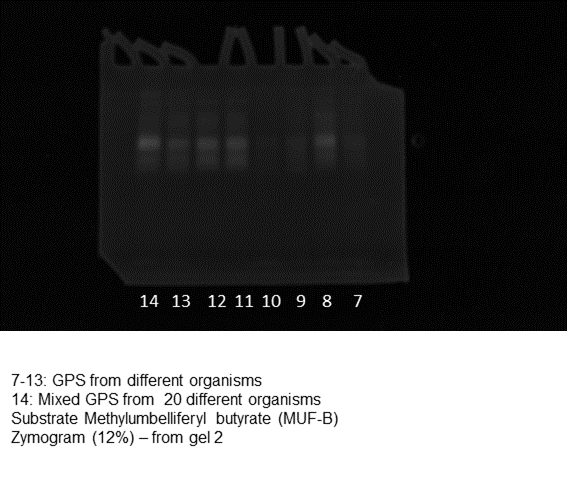

Supplement: Supplemental Information 5 — 7–13= organism ID. 14, jellyfish gastric pouch extract pooled from 20 organisms. [file peerj-08-9794-s005.png]

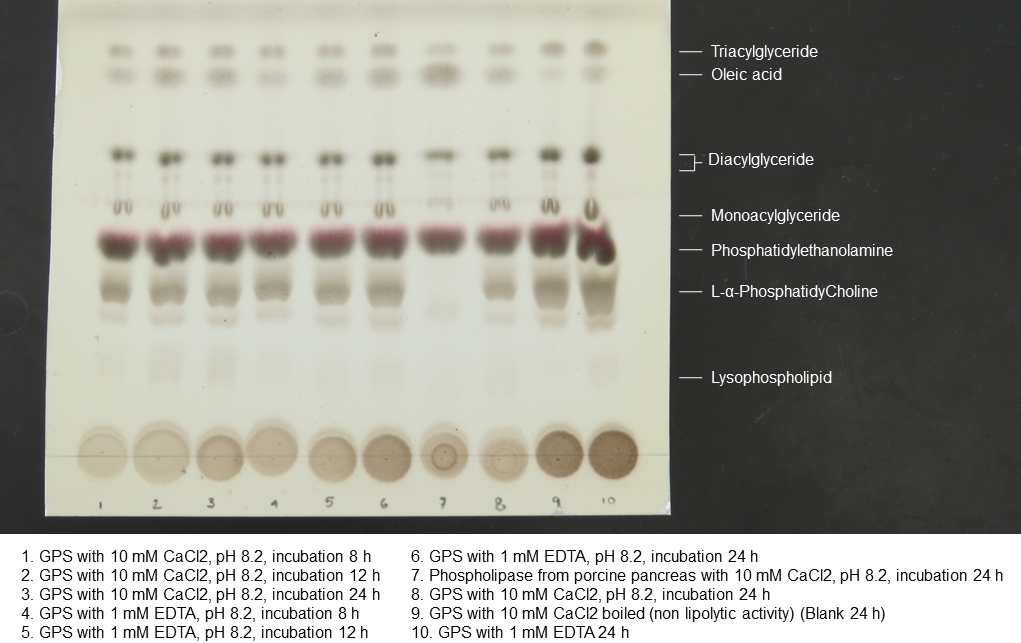

Supplement: Supplemental Information 6 — Imagen shows reagents, and pH/time conditions used during lipolysis. [file peerj-08-9794-s006.png]
